# Revisiting Tundish Flow Characterization: A Combined Eulerian-Lagrangian Study on the Effects of Dams, Baffles, and Side-Wall Inclination

**DOI:** 10.3390/ma18184392

**Published:** 2025-09-20

**Authors:** Ali Mostafazade Abolmaali, Mohamad Bayat, Venkata Karthik Nadimpalli, Thomas Dahmen, Jesper Hattel

**Affiliations:** Department of Civil and Mechanical Engineering, Technical University of Denmark, 2800 Kongens Lyngby, Denmark; mbayat@dtu.dk (M.B.); vkna@dtu.dk (V.K.N.); jhat@dtu.dk (J.H.)

**Keywords:** tundish, casting, CFD simulation, inclusion removal, heat transfer, sustainability

## Abstract

This study aims to use Computational Fluid Dynamics (CFD) analysis to improve inclusion removal efficiency in tundishes used in the steelmaking industry, with the broader goal of promoting more sustainable steel production and supporting circular economy objectives by producing cleaner steel. Inclusions are non-metallic particles, such as alumina, that enter the tundish with the molten steel and travel through it; if not removed, they can exit through the nozzles and adversely affect the mechanical properties of the final product and process yield. An existing tundish design is modified using three passive techniques, including adding a vertical dam, adding a horizontal baffle, and inclining the side walls, to assess their influence on fluid flow behavior and inclusion removal. Residence time distribution (RTD) analysis is employed to evaluate flow characteristics via key metrics such as dead zone and plug flow volume fractions, as well as plug-to-dead and plug-to-mixed flow ratios. In parallel, a discrete phase model (DPM) analysis is conducted to track inclusion trajectories for particles ranging from 5 to 80 μm. Results show that temperature gradients due to heat losses significantly influence flow patterns via buoyancy-driven circulation, changing RTD characteristics. Among the tested modifications, inclining the side walls proves most effective, achieving average inclusion removal improvements of 8% (Case B1) and 19% (Case B2), albeit with increased heat loss due to greater top surface exposure. Vertical dam and horizontal baffle, despite showing favorable RTD metrics, generally reduce the inclusion removal rate, highlighting a disconnect between RTD-based predictions and DPM-based outcomes. These findings demonstrate the limitations of relying solely on RTD metrics for evaluating tundish performance and suggest that DPM analysis is essential for a more accurate assessment of inclusion removal capability.

## 1. Introduction

**Figure 1:**
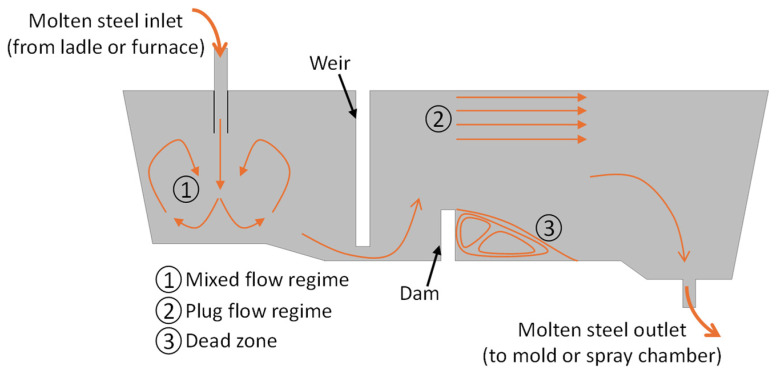
Schematic representation of a typical tundish and associated flow regimes.

A tundish is a buffer vessel commonly used in the steelmaking industry, positioned between the ladle and the continuous casting molds to maintain a nearly constant flow of liquid steel, as schematically illustrated in Figure 1 [1,2,3]. In addition to its role in continuous casting, the tundish also serves as a distributor in a range of steelmaking processes, including mold and ingot casting, spray forming, and gas atomization. In either configuration, a modern tundish is designed not only to regulate the flow of liquid steel but also to perform several important metallurgical functions [4,5]. These include alloy trimming, control of superheat, thermal and chemical homogenization, and most importantly, the removal of non-metallic inclusion particles [1,6,7]. The latter is a critical function, as effective inclusion removal through flotation significantly contributes to achieving high-quality steel production, which is the primary focus of this study.

As sustainability becomes a central objective in modern steelmaking, the demand for cleaner steels has intensified [8,9]. Producing steels with fewer inclusions not only reduces material waste but also extends the service life of high-performance components such as bearings, directly supporting circular economy goals [10]. Simultaneously, the increasing reliance on recycled steel with elevated impurity levels presents new challenges for inclusion control. In this context, optimizing tundish flow behavior is a critical step toward minimizing non-metallic inclusions and enabling more robust, sustainable steel production. As a result, this study revisits tundish flow characterization with a renewed focus on its role in achieving cleaner, more resource-efficient steelmaking.

The primary mechanism of inclusion removal in a tundish is flotation; whereby non-metallic inclusion particles rise and are subsequently absorbed by the top slag layer. An effective tundish facilitates this process by optimizing the flow pattern of liquid steel, promoting the surface-directed flow, and suppressing excessive turbulence from the incoming stream. To this end, various flow-control devices (FCDs), including weirs, dams, baffles, and turbulence inhibitors, as well as designs that promote swirling flow such as the split swirling flow tundish [11], have been introduced as passive yet effective tools to improve tundish performance, particularly in terms of inclusion removal efficiency [4,12,13]. Several studies have investigated how these devices affect fluid flow, temperature distribution, and steel cleanliness, both under steady and transient thermal conditions [14]. Moreover, the positive effect of tundish wall inclination on fluid flow and residence time distribution characteristics was first investigated numerically by He and Sahai [15], who demonstrated that inclining the tundish walls by only 4° from the vertical significantly alters the dominant flow direction inside the tundish compared to the case with vertical walls. More recently, numerical simulations have been employed by various researchers for the optimization of tundish impact zone [16], the design of modified retaining walls and a new ladle shroud for a four-strand tundish [17], the development and optimization of an impact pad for a six-strand tundish [18], and the parameter optimization of a diversion wall in an eight-strand tundish [19]. These studies underscore both the central role of CFD simulations in tundish research and the fact that this remains an active and evolving area of investigation.

To evaluate the performance of various FCD configurations as well as wall inclination, three primary numerical approaches are commonly employed: (1) residence time distribution (RTD) analysis, which characterizes the flow behavior using tracer studies [13,20,21]; (2) Lagrangian particle tracking via discrete phase modeling (DPM), which simulates the trajectories of inclusions [22]; and (3) solving the inclusion transport equation, where the terminal rising velocity of inclusion particles are added to the vertical component of the velocity field [23,24]. These methods provide complementary insights into flow behavior and inclusion transport dynamics in tundish systems.

Residence time is defined as the duration a fluid element spends within the tundish before exiting through the nozzle. Since different elements of the molten steel flow path experience varying residence times, this results in a distribution known as the residence time distribution. The RTD is typically represented as a C-curve, a plot of tracer concentration versus time. To obtain this curve, either experimentally or numerically, a tracer with a specified concentration is injected into the inlet stream at a specific time, and its concentration is monitored at the outlet over time [4,25]. After normalizing the time and concentration with respect to the theoretical mean residence time and the mean tracer concentration, a modified mixed flow model is applied to analyze the RTD curves [26]. Accordingly, the tundish volume is divided into three distinct regions, as shown in Figure 1: the dead zone volume (*V_d_*), the plug flow volume (*V_p_*), and the mixed flow volume (*V_m_*) [4,26]. These volume fractions serve as indicators of tundish performance and are widely used to assess the effectiveness of different FCD configurations. For example, Ahuja and Sahai [26] proposed that minimizing the dead volume and maximizing the plug-to-dead and plug-to-mixed volume ratios are critical for maximizing inclusion removal. Tkadleckova et al. [27] designed an impact pad, a refractory device placed on the tundish floor beneath the ladle shroud to reduce the kinetic energy of incoming molten steel, that increased the plug flow fraction, enhancing the inclusion removal efficiency by up to 15%. Yazdi et al. [28] have mentioned that enhanced inclusion removal can be correlated with larger plug flow fractions and reduced dead zones. Sheng and Zou [29] demonstrated that the removal of larger inclusions (>50 μm) can be qualitatively inferred from RTD curves and proposed the ratio of mixed to plug flow volumes as a new criterion for evaluating inclusion removal. As a result, RTD analysis has become an integral part of tundish flow studies, widely used to optimize the placement and design of FCDs [13,30,31]. For instance, Chen et al. [32] conducted a physical and mathematical modeling study using a 0.4-scale water model to optimize the location and height of a weir and a dam with drain holes in a single-strand tundish. The objective was to identify the configuration with the lowest dead volume and the highest plug flow volume, which was considered the optimal design for inclusion removal. However, despite its usefulness, RTD analysis has inherent limitations, it offers only a qualitative and indirect assessment of inclusion behavior and fails to account for particle-specific dynamics such as variation in size, shape, and density. Therefore, RTD metrics alone may not be sufficient to fully predict or evaluate the inclusion removal efficiency of a tundish system.

To address the limitations of the RTD method and the challenges of directly measuring inclusion separation in experiments, several researchers have employed Lagrangian particle tracking also known as DPM analysis to track inclusion trajectories in the tundish [33]. Sheng [34], using an Eulerian-Lagrangian approach with one-way coupling, studied inclusion behavior in a single-strand tundish and attributed improved removal efficiency to reduced dead volume across four different tundish designs. Zhang et al. [35] reported that higher casting speeds and faster inlet temperature drops reduce inclusion removal efficiency, though they did not establish a link to RTD metrics. More recently, Ding et al. [24] attempted to correlate RTD curves with inclusion behavior through transport equation simulations, concluding that the removal rate does not vary monotonically with RTD parameters such as dead zone, plug flow, and mixed flow volume fractions. They emphasized that a lower dead volume fraction is not sufficient on its own to ensure improved inclusion removal, as structural parameters can also be optimized and the ratio of plug to mixed flow volumes further investigated to enhance removal efficiency.

Another important parameter affecting tundish performance is the temperature variation arising from heat losses or thermal fluctuations in the inlet flow, which has been investigated by several researchers [36,37,38]. Chatterjee and Chattopadhyay [22,39] reported that when there is a temperature difference between the inlet stream and the molten steel inside the tundish (step input), the flow pattern is significantly altered at locations far from the inlet due to buoyancy-driven natural convection, which in turn modifies the temperature distribution and inclusion trajectories. Sheng and Jönsson [40] demonstrated that increasing heat loss from the top surface reduces the mean residence time, leading to larger dead zones and smaller plug flow volume fractions. More recently, Gutiérrez et al. [41] carried out a CFD study highlighting the importance of non-isothermal analysis in tundish investigations. Their results showed that while isothermal simulations may adequately represent flow behavior when fluid dynamics are strongly governed by FCDs, systems without FCDs (or with only weak FCD influence) require non-isothermal modeling for accurate prediction.

The literature review highlights that understanding fluid flow and inclusion behavior in tundishes remains an active area of research. Due to the inherent difficulties of conducting experiments with molten steel, numerical modeling techniques have gained significant attention. Among these, RTD analysis and Lagrangian particle tracking have been widely used to characterize tundish performance, particularly in relation to inclusion removal efficiency.

In this study, the CFD approach is employed to investigate tundish performance with three primary objectives: The first is to identify passive strategies for enhancing inclusion removal rate (IRR) through the use of flow control devices and geometric modifications, such as the addition of vertical dams and horizontal baffles, as well as implementing constant and variable side-wall inclinations. The second objective is to establish a correlation between RTD-based metrics and DPM analysis results. Finally, the influence of isothermal versus non-isothermal conditions on flow behavior and RTD characteristics are examined. It is anticipated that the findings of this study will assist tundish designers in gaining a deeper understanding and more accurate interpretation of RTD curves, ultimately contributing to the development of tundish designs with improved IRR.

## 2. Mathematical Model Description

In this study, ANSYS Fluent (Version 2023 R2) is used to perform CFD simulations. A three-dimensional set of the Navier-Stokes equations is employed to model the continuous phase flow, while the Lagrangian particle tracking using DPM is applied to track the trajectories of inclusions of varying sizes. Additionally, RTD analysis is conducted by introducing a tracer instantaneously at the inlet. Due to the complexity of fluid flow and heat transfer in the tundish, the following assumptions and simplifications are made:

1. The molten steel flow is considered as a single-phase, non-isothermal, steady-state, and incompressible Newtonian fluid.
2. The realizable *k-ε* turbulence model is employed to account for turbulence effects.
3. The free surface is assumed to be flat and subject to zero shear stress.
4. The Boussinesq approximation is adopted to model the natural convection induced by temperature gradients in the tundish.

It should also be noted that, although a tundish operation cycle consists of various stages, this study focuses solely on the intermediate stage between filling and emptying. During this stage, the inlet and outlet mass flows are equal, and the molten steel height in the tundish remains relatively constant. Therefore, a steady-state analysis is justified. In the case of the tundish considered in this study, the steady-state phase represents the majority of the operation. As such, the transient processes during the filling and emptying stages can be neglected without loss of generality.

### 2.1. Continuous Phase Model

The governing equations of motion, including the conservation of mass, momentum, and energy, are solved to simulate the molten steel flow characteristics, as presented below:

(1) $$\frac{\partial u_{j}}{\partial x_{j}}=0,$$

(2) $$\rho_{0}u_{j}\frac{\partial u_{i}}{\partial x_{j}}=-\frac{\partial p}{\partial x_{i}}+\frac{\partial }{\partial x_{j}}\left[\left(\mu +\mu_{t}\right)\left\{\frac{\partial u_{i}}{\partial x_{j}}+\frac{\partial u_{j}}{\partial x_{i}}\right\}\right]-\rho_{0}g_{i}\beta (T-T_{0}),$$

(3) $$\rho_{0}\frac{\partial u_{j}T}{\partial x_{j}}=\frac{\partial }{\partial x_{j}}\left[\left(\frac{\mu }{Pr}+\frac{\mu_{t}}{{Pr}_{t}}\right)\frac{\partial T}{\partial x_{j}}\right],$$

where *ρ_0_* is the density at a reference temperature *T_0_*; and *µ*, *µ_t_*, *β*, *Pr*, and *Pr_t_* represent the dynamic viscosity, turbulent viscosity, thermal expansion coefficient, Prandtl number, and turbulent Prandtl number (taken as 0.85), respectively. The turbulent viscosity is calculated by the following equation [42]:

(4) $$\mu_{t}=\frac{C_{\mu }\rho k^{2}}{\varepsilon },$$

where *k* and *ε* stand for the kinetic energy of turbulence and the rate of turbulence energy dissipation, respectively. Here, the realizable *k-ε* model is utilized to calculate the turbulence variables [43]:

(5) $$\frac{\partial }{\partial t}\left(\rho k\right)+\frac{\partial }{\partial x_{j}}\left(\rho ku_{j}\right)=\frac{\partial }{\partial x_{j}}\left[\left(\mu +\frac{\mu_{t}}{\sigma_{k}}\right)\frac{\partial k}{\partial x_{j}}\;\right]+G_{k}+G_{b}-\rho \varepsilon -S_{k},$$

(6) $$\begin{array}{cc} \frac{\partial }{\partial t}\left(\rho \varepsilon \right)+\frac{\partial }{\partial x_{j}}\left(\rho \varepsilon u_{j}\right) & \\ & =\frac{\partial }{\partial x_{j}}\left[\left(\mu +\frac{\mu_{t}}{\sigma_{\varepsilon }}\right)\frac{\partial \varepsilon }{\partial x_{j}}\;\right]+\rho C_{1}S\varepsilon -\rho C_{2}\frac{\varepsilon^{2}}{k+\sqrt{\nu \varepsilon }}+C_{1\varepsilon }\frac{\varepsilon }{k}C_{3\varepsilon }G_{b}+S_{\varepsilon }, \end{array}$$

where *G_k_*, and *G_b_* represent the generation of turbulence kinetic energy due to mean velocity gradients and buoyancy, respectively. Moreover, *S* and *ν* stand for modulus of mean rate-of-strain tensor and kinematic viscosity while *S_k_* and *S_ε_* are representative of user-defined source terms. Moreover, *σ_k_* and *σ_ε_* are the turbulent Prandtl numbers for *k* and *ε*, respectively. The applicability of the realizable *k-ε* model in tundish flow studies has already been demonstrated by several researchers [13,19,29,35,40,41,44,45], owing to its superior performance in modeling flows involving rotation, boundary layers under adverse pressure gradients, and flow separation. Therefore, detailed information is not provided here for simplicity; further details can be found in References [43,46].

### 2.2. Tracer Dispersion and RTD Analysis

The dispersion of a tracer within the tundish due to an addition of it at the inlet can be modeled by solving a transient passive scalar equation which is of the following form:

(7) $$\frac{\partial }{\partial t}\left(\rho C\right)+\frac{\partial }{\partial x_{j}}\left(\rho u_{j}C\right)=\frac{\partial }{\partial x_{j}}\left(\Gamma_{eff}\frac{\partial C}{\partial x_{j}}\right),$$

where *C* is the concentration (expressed as mass fraction) of the tracer and *Γ_e__ff_* is the effective diffusion mass coefficient. The diffusion coefficient can be related to the effective viscosity (*μ_eff_* = *μ* + *μ_t_*) using the turbulent Schmidt number (*Sc_t_* ~ 1) as follows:

(8) $$Sc_{t}=\frac{\mu_{eff}}{\Gamma_{eff}}.$$

The concentration of tracer at the outlet (*C_out_*) is tracked over time to obtain the C-curve which shows the dimensionless outlet concentration (*C_r_*) as a function of dimensionless time (*θ*) defined as follows:

(9) $$C_{r}=\frac{C_{out}}{C_{avg}},$$

(10) $$\theta =\frac{t_{a}}{t_{r,th}},$$

where *C_avg_* is the spatially averaged concentration of the tracer, *t_a_* is the actual simulation time, and *t_r,th_* is the theoretical mean residence time, which is simply defined as the volume of the tundish divided by the inlet volume flow rate. The RTD analysis is based on the C-curve in order to calculate the volume fraction of different flow regimes in the tundish including dead volume fraction (*V_d_*/*V*), plug flow fraction (*V_p_*/*V*), and mixed flow fraction (*V_m_*/*V*) as follows, using the so-called modified mixed flow model [4,47]:

(11) $$\frac{V_{d}}{V}=1-\frac{Q_{a}}{Q}\theta_{avg},$$

(12) $$\frac{V_{p}}{V}=\frac{\theta_{min}+\theta_{peak}}{2},$$

(13) $$\frac{V_{m}}{V}=1-\frac{V_{d}}{V}-\frac{V_{p}}{V}.$$

In the above equations, *Q_a_*/*Q* is the fractional volumetric flow rate through the active region being equal to the area under the C-curve from *θ* = 0 to *θ* = 2. Moreover, *θ_min_* and *θ_peak_* are the dimensionless times of minimum and peak concentration at the tundish outlet, respectively, while *θ_avg_* is equal to the mean dimensionless residence time up to *θ* = 2 and is calculated by the following equation:

(14) $$\theta_{avg}=\frac{\left(\frac{\int_{\theta =0}^{\theta =2}tCdt}{\int_{\theta =0}^{\theta =2}Cdt}\right)}{t_{r,th}}.$$

### 2.3. Discrete Phase Model (DPM)

The trajectory of the inclusion particles in the molten steel can be predicted by integrating the force balance on the particles, which is described in a Lagrangian reference frame. This force balance relates the particle’s inertia to the various forces acting upon it and can be expressed as [46]:

(15) $$m_{p}\frac{d{\vec{u}}_{p}}{dt}=m_{p}\frac{18\mu C_{d}Re}{{24\rho }_{p}d_{p}^{2}}\left(\vec{u}-{\vec{u}}_{p}\right)+m_{p}\frac{\vec{g}(\rho_{p}-\rho )}{\rho_{p}}+{\vec{F}}_{p}+{\vec{F}}_{vm}+{\vec{F}}_{l}+{\vec{F}}_{td},$$

where *m_p_*, *ρ_p_*, and *d_p_* represent the mass, density, and diameter of particles, respectively, while *u_p_* is the particle velocity and *u* stands for the continuous phase velocity. The first and second terms on the right-hand side of the equation show the drag and buoyancy forces, respectively. Other forces include:

- Pressure gradient force (*F_p_*): the force exerted on a particle due to spatial changes in fluid pressure.
- Virtual mass force (*F_vm_*): an added force accounting for the acceleration of surrounding fluid when a particle accelerates.
- Saffman’s lift force (*F_l_*): a lift force due to the velocity gradient perpendicular to the particle motion in a shear flow.
- Turbulent dispersion force (*F_td_*): a force acting on a particle due to the effect of turbulent eddies causing random scattering and enhanced mixing.

A detailed description of these forces and their governing equations can be found in [48] and is omitted here for the sake of brevity. The relative Reynolds number (*Re*) is defined as Equation (16) while the drag coefficient (*C_d_*) of the particle is modeled using the spherical drag law as shown in Equation (17):

(16) $$Re=\frac{\rho d_{p}\left|{\vec{u}}_{p}-\vec{u}\right|}{\mu },$$

(17) $$C_{d}=a_{1}+\frac{a_{2}}{Re}+\frac{a_{3}}{Re^{2}},$$

where *a_1_*, *a_2_*, and *a_3_* are constants that are given by Morsi and Alexander [49] based on an experimental drag–Reynolds number relationship for spherical particles. The Reynolds number range from 0.1 to 5 × 10^4^ is divided into seven intervals, and these constants are determined for each interval. It should also be emphasized that coagulation forces are not included in the present model, following the conclusions of References [50,51,52], which indicate that hydrodynamic forces are the primary factors governing inclusion trajectories and removal efficiency rather than particle–particle interactions.

### 2.4. Description of Simulated Cases

This study focuses on a single-strand tundish from a specific steel plant to examine how vertical dams, horizontal baffles, and the side-wall inclination affect inclusion removal efficiency. The analysis is performed using RTD curves and the Lagrangian particle tracking using DPM. The findings of this study can be applied to tundishes used across various steelmaking processes, including continuous casting, mold and ingot casting, spray forming, and gas atomization.

**Figure 2:**
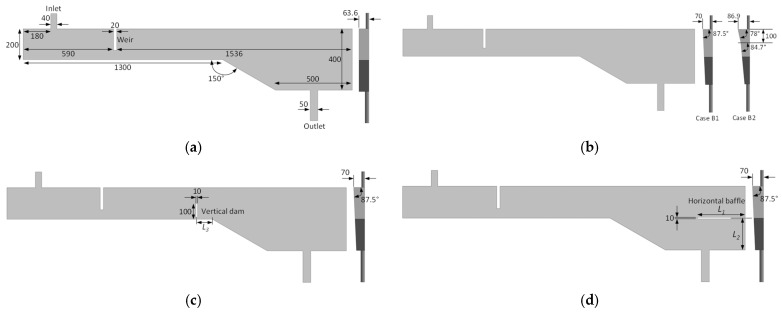
Dimensions of different tundishes studied (unit: mm), (**a**) Case A, (**b**) Case B, (**c**) Case C, and (**d**) Case D. Detailed geometric characteristics of Cases A to D can be found in Table 1.

The original tundish configuration includes a single weir and vertical side walls (i.e., with no inclination), as illustrated in Figure 2a. To study the influence of various flow-control devices (FCDs) and side wall angles, four groups of tundish configurations were developed, labeled as Cases A through D:

- Case A: The original tundish with one weir and vertical side wall (1 case).
- Case B: Tundish configurations with inclined side walls and without dams and baffles (2 cases).
- Case C: Tundish configurations with a vertical dam and inclined side wall (2 cases).
- Case D: Tundish configurations with a horizontal baffle and inclined side wall (15 cases).

Cases B1 and B2 were designed to investigate the effect of side wall inclination on tundish performance with respect to inclusion removal. Case B1 features a side wall inclined at a constant angle, whereas Case B2 incorporates a variable-angle inclined side wall. Cases C were developed to study the influence of vertical dam location on tundish performance. Cases D were aimed at evaluating the impact of both the position and length of horizontal baffles on the performance of the tundish. In total, 20 distinct cases were examined. Their dimensions are depicted in Figure 2, and detailed geometric characteristics are provided in Table 1. It should be noted that all cases have identical volumes to maintain the same theoretical residence time.

### 2.5. Boundary Conditions and Calibration Process

**Figure 3:**
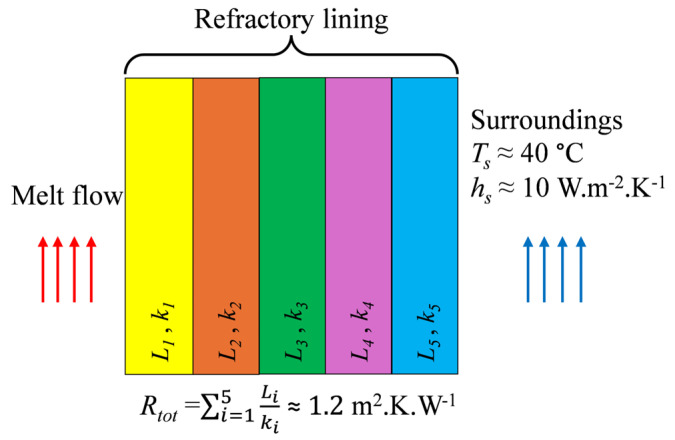
Tundish refractory lining and thermal boundary conditions.

A half-model of the tundish was used in all cases by taking advantage of the symmetry plane. The following boundary conditions were applied to solve the governing equations for the continuous phase. A constant mass flow rate of 50 kg/min was specified at the inlet, while a zero-gauge pressure condition was imposed at the outlet. All walls were treated with a no-slip boundary condition, except for the top surface, where a zero-shear condition was applied to represent the presence of a slag layer. As shown in Figure 3, heat loss from all side and bottom walls was modeled as a combination of conduction heat transfer through the refractory lining, composed of five different layers, and convection heat transfer to the surrounding air on the outer surface. Heat loss from the top surface was modeled as pure thermal radiation.

To ensure a validated heat transfer model, the convection heat transfer coefficient at the side walls (*hₛ*) and the emissivity at the top surface (*ε_top_*) were determined through calibration against industrial measurements obtained during steady-state tundish operation. The tundish typically reaches steady-state conditions within three minutes, and these conditions are maintained for a duration of approximately 30–40 min. The relevant temperature data included:

1. Melt temperature at the inlet (measured by immersion thermocouple, ~1550 °C).
2. Temperature of the tundish cover walls under nitrogen protection (measured with a handheld infrared thermometer, ~200 °C).
3. Outer surface temperatures of the tundish side, front, and back walls (measured with a handheld infrared thermometer, ranging between 60 °Cand 90 °C).
4. Melt temperature at the outlet nozzle (measured by high-temperature pyrometer, ~1530 °C).

The calibration process was conducted as follows:

A CFD model was run with an inlet melt temperature of 1550 °C (measurement No. 1). The top surface was modeled as a radiative boundary with emissivity *ε_top_* and a wall temperature of 200 °C (measurement No. 2). Side walls were modeled with convective heat transfer to an ambient air temperature of 40 °C (average measured surrounding temperature). The convection heat transfer coefficient (*hₛ*) was estimated to be approximately 10 W·m^−2^·K^−1^, based on measured side wall temperatures (measurement No. 3), ambient air temperature, and Nusselt number correlations for natural convection (Churchill and Chu [53]).

The steady-state outlet melt temperature predicted by the CFD model was compared with the measured outlet temperature (~1530 °C, measurement No. 4). By varying the emissivity, it was determined that *ε_top_* ≈ 0.1 provided good agreement between the predicted and measured outlet temperatures. These calibrated values (*hₛ* ≈ 10 W·m^−2^·K^−1^, *ε_top_* ≈ 0.1) were then applied consistently in all subsequent simulations.

### 2.6. Numerical Procedure (Tracer and DPM Analysis)

After computing the steady-state flow field, the solution was used as the basis for a transient simulation in which only the passive scalar equation was solved. To model the tracer injection, zero mass flux was applied at all walls except at the inlet boundary, where a constant mass fraction of 1 was imposed for a duration of 2 s (from *t* = 0 to *t* = 2 s). After *t* > 2 s, the inlet mass fraction was set to zero. To derive the RTD curves, the tracer mass fraction at the outlet was monitored from the beginning up to *t* = 1000 s. Considering the theoretical residence time (*t_r,th_*) of the tundishes, which is approximately 300 s, this simulation duration ensures that the dimensionless time exceeds 3.0, which is sufficient for determining different volume fractions of the tundishes using Equations (11)–(14).

The one-way DPM analysis was performed after the continuous phase reached a steady-state condition. Various particle diameters including 5, 10, 15, 20, 30, 40, 50, 60, 70, and 80 μm were injected separately through the inlet. Each injection consisted of 2000 alumina particles with a density of 3960 kg/m^3^. The top surface and outlet boundaries were set to trap and escape, respectively, while all other walls in the domain were assigned a reflect boundary condition. Agglomeration and collision between inclusion particles were not considered in the present analysis.

### 2.7. Mesh Generation

A polyhedral volume mesh with an element size of 6 mm was generated using Fluent Meshing. This mesh size ensured that the RTD curve and temperature measurements were mesh independent. To better capture near-wall physics, inflation layers were created on all walls. The mesh was refined near the inlet, outlet, and FCDs to accurately represent curvature. The total number of mesh elements across different cases ranged from 334,000 to 396,000.

### 2.8. Solution Strategy and Convergence Criteria

The Semi-Implicit Method for Pressure-Linked Equations (SIMPLE) algorithm was used to solve the discretized equations of motion for the continuous phase. The effect of hydrostatic pressure was taken into account in the simulations. A second-order upwind scheme was applied to compute the convective fluxes in the momentum, turbulent kinetic energy, turbulent dissipation rate, and energy equations, while the PRESTO! (PREssure STaggering Option) algorithm was employed to solve the pressure correction equation for improved convergence. The steady-state solution was considered converged when the residuals of all solved variables fell below 10^−6^. The converged solution was then used to solve the transient tracer dispersion equation with a time step size of 0.5 s. It was also used to calculate the trajectory of inclusion particles using steady-state DPM analysis.

## 3. Model Validation

To validate the mathematical model, two previous studies involving water model experiments were examined. First, the experimental work by Sheng and Chen [13] on a scaled-down, isothermal water model of a single-strand tundish equipped with a weir and a dam was used to compare the RTD curves obtained from CFD simulations with those derived experimentally. Subsequently, the non-isothermal water model experiment conducted by Sheng et al. [54] on a single-strand slab caster tundish was employed to compare CFD results with experimental data in terms of temperature distribution and heat transfer characteristics.

**Figure 4:**
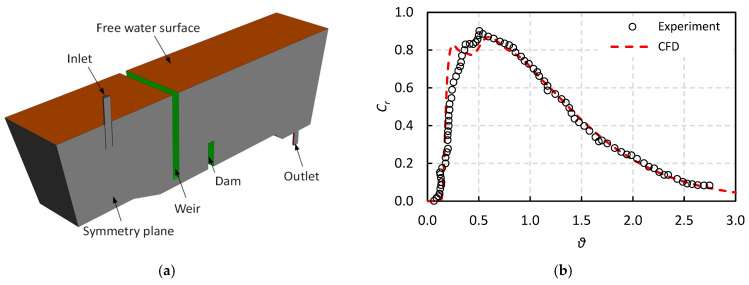
Comparison of isothermal water model experiment of Sheng and Chen [13] with present CFD model: (**a**) Model geometry, (**b**) RTD curve.

The geometry of the single-strand tundish used in Ref. [13] is illustrated in Figure 4a, where half of the tundish was modeled using a symmetry plane. Figure 4b presents the comparison between the CFD-simulated and experimentally obtained RTD curves, showing very good agreement. Specifically, the CFD results accurately predict key parameters such as the breakthrough time (i.e., the time at which the tracer is first detected at the outlet), the peak time, the peak concentration, and the slope of the RTD curve following the peak. These results confirm the validity of the CFD simulation in predicting the RTD behavior.

**Figure 5:**
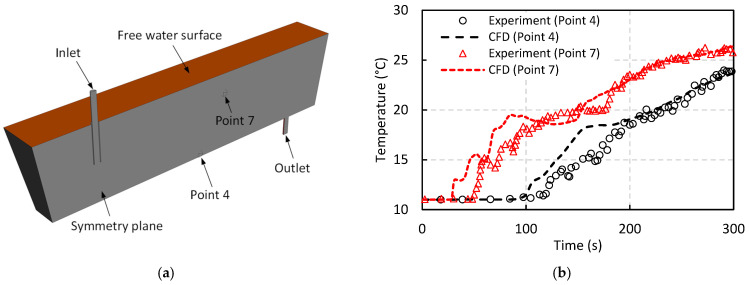
Comparison of non-isothermal water model experiment of Sheng et al. [54] with present CFD model: (**a**) Model geometry, (**b**) Temperature variation at specified points.

Following this, a transient simulation was conducted to compare CFD results with the experimental data of Sheng et al. [54] under non-isothermal conditions in a single-strand tundish, as illustrated in Figure 5a. In this setup, the inlet temperature was 30 °C higher than the initial temperature of the water in the tundish. In the experiment, temperature variations at 11 specified locations were recorded to investigate flow behavior influenced by both natural and forced convection. Figure 5b presents a comparison of the temperature variations over time at two representative points: point 4, located near the tundish bottom, and point 7, near the free surface. While some discrepancies are observed between the CFD and experimental results during the initial stages, these differences become negligible as the simulation progresses and the flow field approaches steady-state conditions. This demonstrates the reliability of the CFD model in predicting the thermohydraulic behavior of the flow under non-isothermal conditions, which is representative of actual tundish operation where significant heat losses result in noticeable temperature drops in molten steel.

## 4. Results and Discussion

The CFD simulation results are presented in five sub-sections. First, Section 4.1 compares the steady-state flow of molten steel under isothermal and non-isothermal conditions for the different cases studied. Section 4.2 presents the RTD curves obtained from tracer dispersion analysis, which are subsequently used for volume fraction evaluation. Section 4.3 provides inclusion tracking results, extending the RTD analysis by applying a DPM to alumina inclusion particles with diameters ranging from 5 to 80 μm. A heat transfer analysis of the tundishes with inclined walls is presented in Section 4.4, followed by a rough economic assessment of the proposed variable-angle side wall design in Section 4.5. Finally, Pearson correlation is employed in Section 4.6 to investigate potential relationships between the RTD-based metrics and the inclusion removal rate (IRR).

### 4.1. Flow Pattern Analysis

Previous studies have shown that non-isothermal modeling provides more accurate predictions of steel cleanliness, flow behavior, and thermal distribution. Therefore, in this study, a comparative analysis of fluid flow patterns under isothermal and non-isothermal conditions is conducted using the CFD model. The non-isothermal condition arises primarily from heat losses, mainly occurring through the top slag layer.

**Figure 6:**
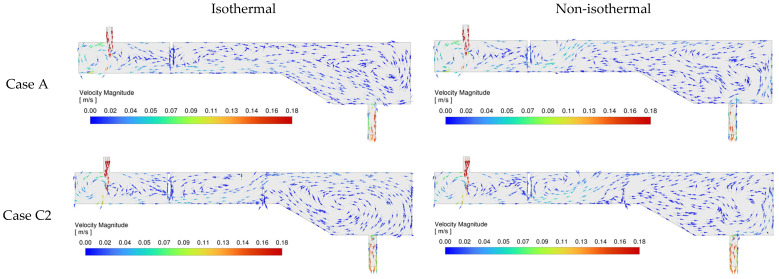
Velocity field at symmetry plane under isothermal and non-isothermal conditions for Case A and Case C2.

Figure 6 illustrates the velocity field distributions of molten steel under both isothermal and non-isothermal conditions for Case A (with a single weir) and Case C2 (featuring one weir and one vertical dam). The velocity vectors in the inlet zone (upstream of the weir) show no significant differences between isothermal and non-isothermal conditions, nor between the two configurations (Case A and Case C2). This indicates that neither buoyancy effects nor the presence of flow control devices such as a vertical dam have a notable impact on the velocity field in the inlet region.

In all cases, once the inlet jet impinges on the tundish bottom, part of the flow ascends along the side walls toward the top free surface, forming a clockwise recirculation zone. Meanwhile, another portion of the flow travels beneath the weir, with the overall flow direction between the inlet and the weir oriented from the inlet toward the weir, as indicated by the velocity vectors. The primary distinction among the different cases emerges in the flow field downstream of the weir.

In Case A, under isothermal conditions, the flow passes beneath the weir and remains near the tundish bottom, moving directly toward the outlet. Subsequently, part of the flow exits through the outlet, while the remaining portion rises along the end wall and reaches the free surface (i.e., the top slag layer). It then flows along the upper surface from the outlet end back toward the weir, completing a circulation loop. A distinct counterclockwise recirculation zone is observed downstream of the weir under these conditions.

In contrast, under non-isothermal conditions, the downstream flow pattern is significantly altered by buoyancy effects. In this scenario, the flow rises immediately after passing the weir and reaches the free surface due to the effect of buoyancy. It then moves along the top surface toward the tundish end wall, where it descends after impingement. A portion of this descending flow exits through the outlet, while another portion ascends again and travels toward the base of the sloped surface. Upon reaching the beginning of the slope, the flow splits: one part adheres to the bottom and returns toward the outlet, while the other part also follows the bottom wall but circulates back toward the weir. Overall, under non-isothermal conditions, the dominant flow circulation downstream of the weir becomes clockwise, although a small counterclockwise vortex is still evident just behind the weir, along with another minor counterclockwise circulation zone above the sloped surface.

In Case C2, under isothermal conditions, the addition of a vertical dam significantly modifies the flow behavior near the outlet. The circulation direction in this region becomes clockwise, contrasting with the counterclockwise pattern observed in the absence of the dam. However, under non-isothermal conditions, the presence of the dam has minimal influence on the overall flow behavior in the downstream region. A dominant clockwise circulation zone persists near the tundish outlet, accompanied by a minor counterclockwise vortex over the inclined surface.

**Figure 7:**
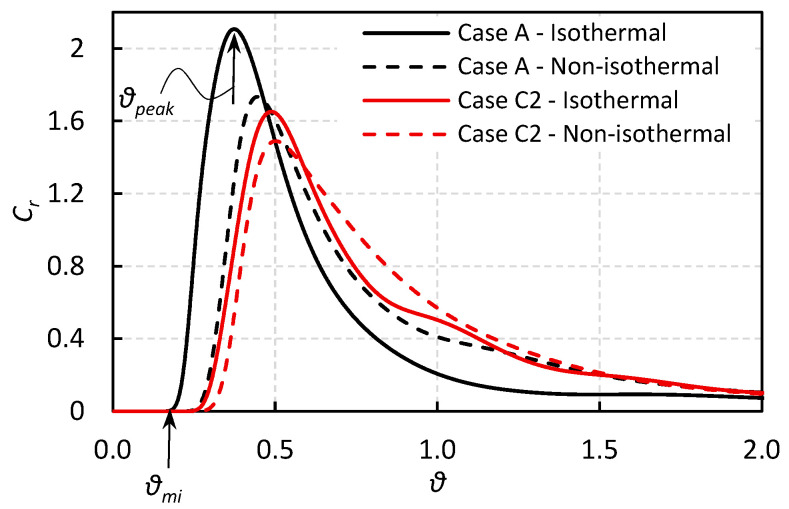
Comparison of RTD curves of Case A and Case C2 under isothermal and non-isothermal conditions. The dimensionless breakthrough time (*θ_min_*) and the dimensionless peak time (*θ_peak_*) for Case A are presented in the graph.

Figure 7 presents the RTD curves for Cases A and C2 under both isothermal and non-isothermal conditions. The RTD curve under isothermal conditions, particularly in Case A, shows a notable difference compared to the RTD obtained under non-isothermal conditions. Specifically, under non-isothermal conditions, the dimensionless breakthrough time (*θ_min_*) is delayed, and the dimensionless time at which the concentration peak occurs (*θ_peak_*) is also increased. Additionally, the slope of the curve after the peak differs, being slightly lower in the non-isothermal case. Another important observation is that the difference between the isothermal and non-isothermal RTD curves is smaller in the presence of the dam than in its absence.

These changes affect the distribution of the volume fractions of various regions within the tundish, including the dead zone, plug flow volume, and mixed flow volume, as shown in Table 2. It is observed that although the theoretical residence time (*t_r,th_*) remains approximately the same across all cases, the mean dimensionless residence time (*θ_avg_*) is higher under non-isothermal conditions compared to the isothermal case. Moreover, both the breakthrough time (*t_min_*) and peak time (*t_peak_*) are greater in non-isothermal conditions. Another important finding is that non-isothermal conditions have a more pronounced effect on the volume fractions in Case A (without a dam) than in Case C2 (with one dam), which is in line with the findings of Gutierrez et al. [41].

Overall, the main conclusion is that non-isothermal conditions can have a significant impact on fluid flow and RTD behavior in tundishes, especially in the absence of a dam. Unlike isothermal analyses, which assume a uniform temperature distribution, non-isothermal modeling accounts for temperature gradients that give rise to buoyancy-driven flows and thermal stratification. These temperature-induced effects alter flow patterns, potentially improving residence time distribution, decreasing the likelihood of short-circuiting, and reducing the volume fraction of dead zones within the tundish. Inclusion removal efficiency may also improve under thermal gradients due to enhanced plug flow and diminished dead zone regions. As a result, all subsequent simulations in this study were conducted under non-isothermal conditions.

### 4.2. RTD and Volume Fraction Analysis

**Figure 8:**
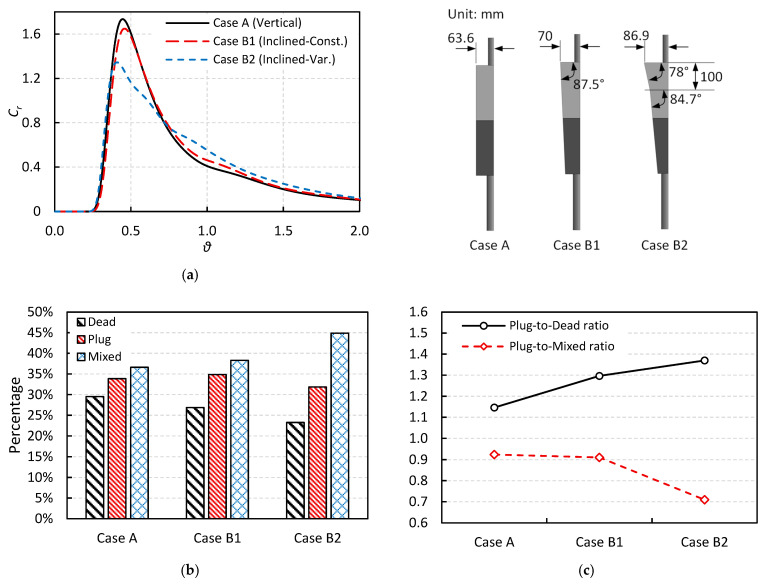
The effect of side wall inclination angle on the: (**a**) RTD curve, (**b**) volume fractions, and (**c**) volume fraction ratios (The upper-right subfigure illustrates the differences in cross-sectional geometry for the various cases, reproduced from Figure 2 for clarity).

The RTD curves for the simulated cases listed in Table 1, along with the volume fraction analysis, are presented in this section. It should be noted that all cases have approximately the same total volume and, consequently, the same theoretical residence time. Figure 8a compares the RTD curves of Cases A, B1, and B2, which are configured with vertical side walls, inclined side walls with a constant angle, and inclined side walls with a variable angle, respectively.

It is observed that all cases exhibit approximately the same breakthrough time. Moreover, while there is no significant difference between the RTD curves of Case A and Case B1, a noticeable drop in both peak concentration and peak time is observed for Case B2. The percentage contributions of different flow regimes including dead zone, plug flow, and mixed flow are shown in Figure 8b. Inclining the side walls has a considerable effect in reducing the percentage of the dead zone, which might be a favorable modification for improving the overall flow regime in the tundish and enhancing inclusion removal efficiency.

Furthermore, it is observed that Case B2, with a variable inclination angle, performs better than Case B1, with a constant inclination angle, in terms of dead zone reduction. However, Case B2 also exhibits a lower plug flow fraction, which is generally considered the most favorable flow regime for inclusion removal. Therefore, it may appear that using a variable inclination angle design (Case B2) could simultaneously have both a positive effect (due to dead zone reduction) and a negative effect (due to a decrease in plug flow fraction). Hence, it is also beneficial to compare the plug-to-dead and plug-to-mixed volume ratios, since higher values of these ratios are known to support better inclusion removal efficiency [4,26]. Figure 8c illustrates these ratios for Cases A, B1, and B2. It is observed that inclining the side walls increases the plug-to-dead volume ratio, while it decreases the plug-to-mixed volume ratio. As a result, it is difficult to draw a definitive conclusion about the inclusion removal efficiency of these modified designs based solely on RTD and volume fraction analyses. Therefore, additional insight can be gained from the DPM analysis, which is discussed in the following sections.

**Figure 9:**
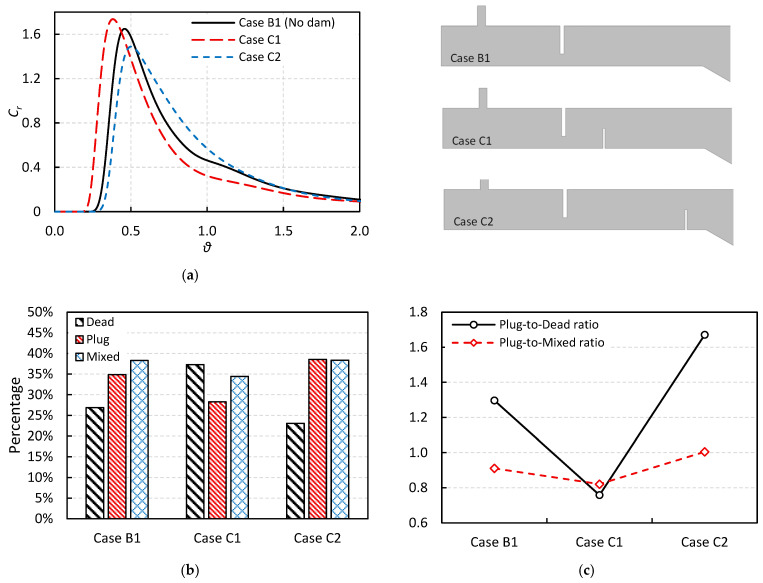
The effect of vertical dam on the: (**a**) RTD curve, (**b**) volume fractions, and (**c**) volume fraction ratios (The upper-right subfigure illustrates the differences in cross-sectional geometry for the various cases, reproduced from Figure 2 for clarity).

Figure 9a illustrates the RTD curves for Case B1, C1, and C2, which correspond to configurations with no dam, a vertical dam located immediately behind the weir, and a vertical dam positioned farther downstream from the weir, respectively. All cases feature a side-wall inclination with a constant angle. Comparing the RTD curves of Cases B1 and C1, it is observed that adding a vertical dam just behind the weir negatively affects the flow behavior by reducing both the breakthrough time and peak time. In contrast, moving the dam farther downstream in Case C2 increases both breakthrough and peak times, which may lead to improved tundish performance.

To further examine the impact of dam placement, the volume fractions of different flow regimes are presented in Figure 9b. Case C1 exhibits an increase in the dead volume and a decrease in the plug flow volume compared to Case B1, both of which can adversely affect inclusion removal efficiency. However, in Case C2, relocating the vertical dam results in a reduction in the dead zone volume fraction and an increase in the plug flow fraction, emphasizing the importance of dam positioning in optimizing flow characteristics.

Additionally, Figure 9c compares the plug-to-dead and plug-to-mixed volume ratios for all three cases. Case C1 exhibits the poorest performance, with the lowest values for both ratios, while Case C2 demonstrates the best performance with the highest values. Based on the RTD and volume fraction analyses, it can be inferred that Case C2 is expected to offer superior inclusion removal performance compared to Cases C1 and B1. This conclusion needs to be assessed by DPM analysis, as will be discussed in the following sections.

**Figure 10:**
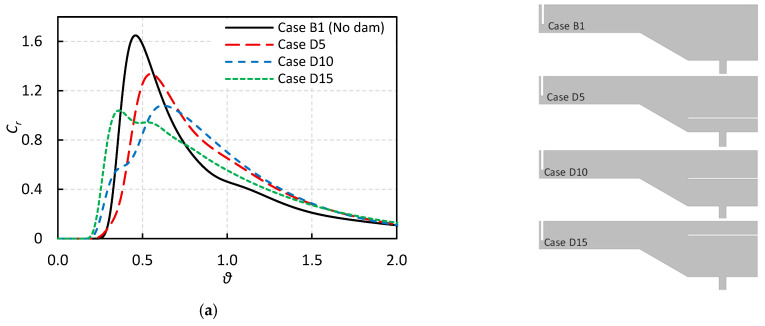

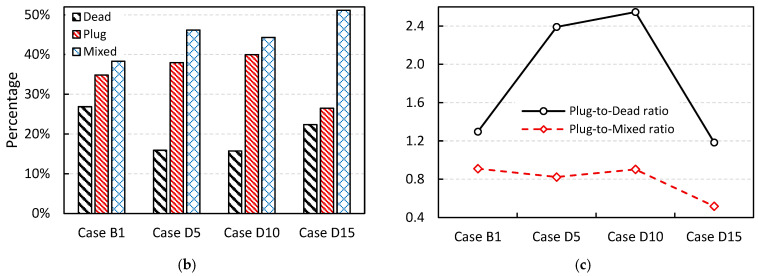
The effect of horizontal baffle on the: (**a**) RTD curve, (**b**) volume fractions, and (**c**) volume fraction ratios (The upper-right subfigure illustrates the differences in cross-sectional geometry for the various cases, reproduced from Figure 2 for clarity).

Figure 10 presents the influence of adding a horizontal baffle to the vertical wall at the end of the tundish on the RTD curves and volume fractions. All cases shown in this figure feature a side-wall inclination with a constant angle. As shown in Table 1, a total of 15 cases equipped with a horizontal baffle are analyzed, categorized into three groups based on the vertical position of the baffle relative to the bottom of the tundish.

- In Cases D1 to D5, the baffle is located 100 mm above the bottom wall.
- In Cases D6 to D10, the baffle is positioned 200 mm above the bottom wall, i.e., at the same level as the starting point of the sloped surface.
- In Cases D11 to D15, the baffle is placed 300 mm above the bottom wall.

Within each group, the length of the horizontal baffle varies from 100 mm to 500 mm in 100 mm increments. Thus, the final case in each group features a horizontal baffle with a length of 500 mm. However, only the cases with the longest baffle in each group are shown in Figure 10 as representatives.

The RTD curves in Figure 10a indicate that the addition of a horizontal baffle slightly decreases the breakthrough time, suggesting an increase in short-circuiting, an undesirable effect. However, the peak time increases when the baffle is located below or at the same level as the sloped surface (i.e., Cases D5 and D10), leading to a higher plug flow volume fraction. In contrast, when the baffle is placed above the sloped surface (Case D15), the peak time also decreases, which can negatively impact tundish performance in terms of inclusion removal.

To better understand the effect of the horizontal baffle on flow regimes, the percentage contributions of various volume fractions are shown in Figure 10b. It is observed that positioning the horizontal baffle below the sloped surface significantly reduces the dead zone volume while increasing the plug flow volume, an outcome generally favorable for enhancing inclusion removal efficiency. However, in Case D15, although the dead zone volume is reduced compared to Case B1 (without horizontal baffle), the plug flow volume is also decreased. Furthermore, Case D15 exhibits a higher dead zone and lower plug flow volume fractions compared to Cases D5 and D10.

It is also useful to compare the plug-to-dead and plug-to-mixed volume ratios, as illustrated in Figure 10c. Adding a horizontal baffle below the sloped surface nearly doubles the plug-to-dead volume ratio, while the plug-to-mixed ratio remains relatively unchanged. On the other hand, placing the baffle above the sloped surface reduces both ratios. Overall, based on the RTD analysis, Case D10 appears to offer the best performance in terms of inclusion removal efficiency among the cases presented in Figure 10. However, this assessment should be further validated by the DPM analysis discussed in the next section.

**Figure 11:**
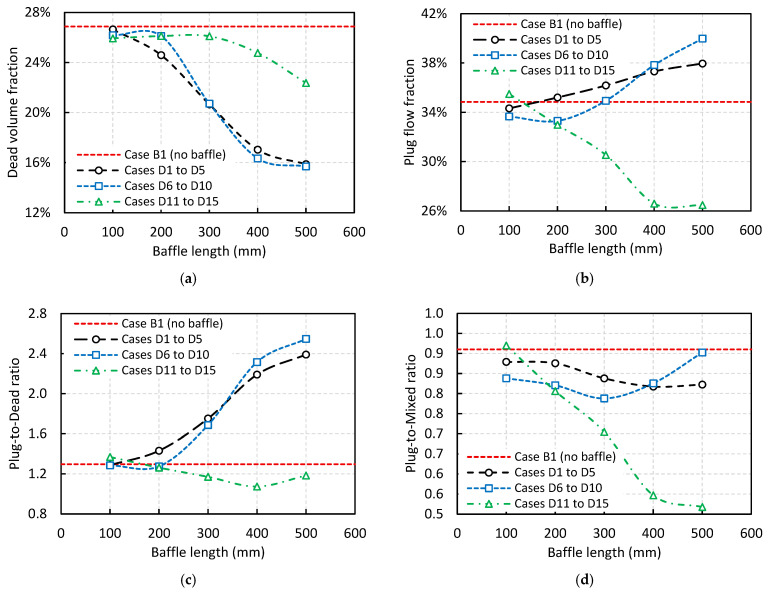
The effect of horizontal baffle length on the: (**a**) dead zone volume, (**b**) plug flow volume, (**c**) plug-to-dead ratio, and (**d**) plug-to-mixed ratio.

To investigate the effect of horizontal baffle length on tundish performance, Figure 11 presents the volume fraction analysis for all cases with a horizontal baffle (Cases D1 to D15). For better comparison, the results of Case B1 (without a horizontal baffle) are also included in all graphs shown in Figure 11. Figure 11a reveals that the addition of a horizontal baffle reduces the dead zone volume, regardless of its length or position. Moreover, it shows that longer baffles positioned below the sloped surface have a greater impact on reducing the dead zone volume.

Figure 11b illustrates that the addition of a horizontal baffle can potentially increase the plug flow fraction, although this effect depends on the baffle’s vertical location. Specifically, when the baffle is positioned above the sloped surface (Cases D11 to D15), it tends to reduce the plug flow fraction. In contrast, when the baffle is located below or at the same level as the start of the sloped surface, it can increase the plug flow fraction, though this is also dependent on baffle length. For instance, in the latter configuration, when the length is shorter than 300 mm there is no improvement in plug flow fraction, while longer baffles (>300 mm) result in a noticeable increase.

To gain a clearer understanding, the plug-to-dead volume ratio is shown in Figure 11c. This ratio indicates that to achieve improvement, the baffle should not be placed above the sloped surface, and it should not be too short. Another important parameter is the plug-to-mixed volume ratio, shown in Figure 11d. It is evident that the addition of a horizontal baffle reduces this ratio in almost all cases (except Case D11), which may negatively impact tundish performance with respect to inclusion removal efficiency.

### 4.3. DPM Analysis

**Figure 12:**
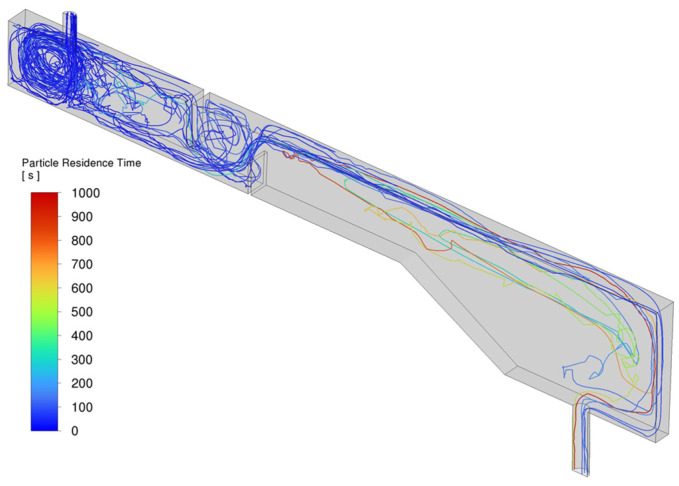
The trajectories of 20 particles (50 μm diameter) in Case C1, color-coded by residence time in the tundish. Six particles exited through the outlet, while fourteen were captured by the top slag layer.

As mentioned in Section 2.4, for each of the considered particle diameters (5, 10, 15, 20, 30, 40, 50, 60, 70, and 80 μm), 2000 alumina particles (density: 3960 kg/m^3^) were injected separately through the inlet to model the trajectories of inclusion particles in the tundish. It is worth mentioning that, to represent the worst-case scenario for inclusion removal, alumina was chosen as the model inclusion in this study due to its highest density and consequently the lowest buoyancy among common inclusions such as CaO and SiO_2_. Particles that reach the top surface are assumed to be trapped by the slag layer, removed from the domain, and counted in the calculation of the removal rate using Equation (18):

(18) $$Removal\;rate=\frac{N_{total}-N_{trapped}}{N_{total}}\times 100,$$

where *N_total_* and *N_trapped_* are the total number of injected particles and the number of particles trapped by the top slag layer, respectively. The remaining particles, which are not trapped by the slag layer, eventually exit the domain through the outlet after rebounding from the walls and are therefore considered as escaped inclusions. To provide a representative overview of typical DPM analysis results, Figure 12 shows the trajectories of 20 sample particles (out of a total of 2000 tracked particles) with a diameter of 50 μm in Case C1. The trajectories are color-coded based on the particles’ residence time in the tundish. Among the 20 particles, six exited through the outlet, while the remaining fourteen were trapped by the top slag layer.

**Figure 13:**
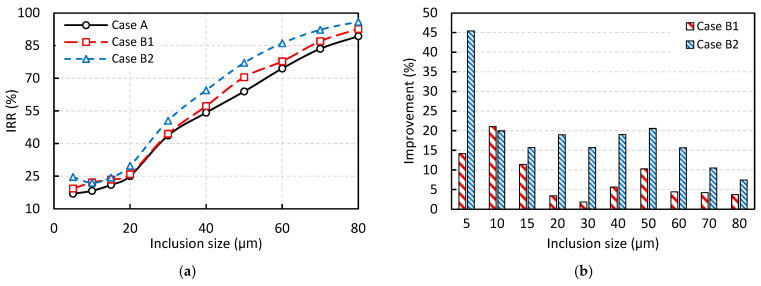
The effect of side wall inclination angle on inclusion removal rate (IRR) based on DPM analysis: (**a**) IRR, and (**b**) Percentage improvement in IRR compared to the base case with vertical side walls (Case A).

Figure 13 presents the results of the DPM analysis for Cases A, B1, and B2, illustrating the effect of side-wall inclination on the IRR. The geometric differences between these cases were previously described in Figure 8. As shown in Figure 13a, the removal rate increases with particle size in all cases. This trend is primarily attributed to the stronger buoyancy force acting on larger particles, which facilitates their flotation to the slag layer. For inclusion particles smaller than 20 μm, the increase in removal rate with particle size is relatively modest. However, for particles larger than 20 μm, a more substantial improvement is observed. For instance, in Case A with vertical side walls, increasing the particle diameter from 5 μm to 20 μm, results in a removal rate increase from 17% to 25%, a 1.47-fold improvement despite a fourfold increase in diameter. In contrast, increasing the particle size from 20 μm to 80 μm leads to an increase from 25% to 89%, representing a 3.56-fold improvement.

Another key observation is that inclining the side walls positively affects inclusion removal, as both Cases B1 and B2 show higher removal rates compared to Case A. To better illustrate this effect, Figure 13b presents the percentage improvement in IRR for Cases B1 and B2 relative to Case A as a function of inclusion size. The results indicate that wall inclination enhances removal rate across all inclusion sizes. Moreover, the variable inclination angle used in Case B2 outperforms the constant inclination angle in Case B1 in almost all inclusion sizes. On average, assuming equal weighting across all inclusion sizes, Cases B1 and B2 improve IRR by approximately 8% and 19%, respectively. This improvement can primarily be attributed to the increased free surface area available in tundishes with inclined side walls, which facilitates particle flotation, while maintaining the same overall tundish volume and theoretical residence time. However, this improvement comes at the cost of increased heat loss, which will be discussed in the following section.

**Figure 14:**
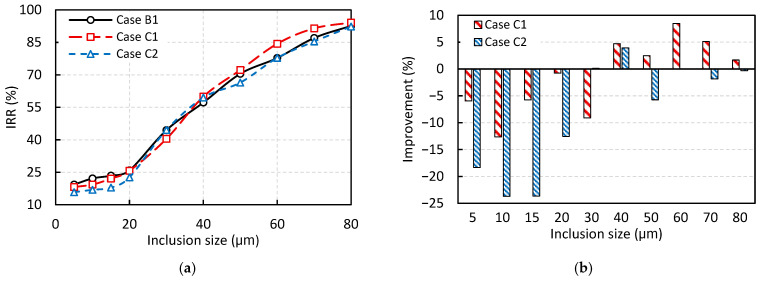
The effect of the vertical dam on inclusion removal rate (IRR) based on DPM analysis: (**a**) IRR, and (**b**) Percentage improvement in IRR compared to the base case without dam (Case B1).

Figure 14 presents the effect of adding a vertical dam to the tundish on the IRR, based on the DPM analysis. As shown in Figure 14a, the overall trend in removal rate is consistent with that observed in Figure 13a, exhibiting an increase in removal rate with inclusion size, an effect that becomes more pronounced for particles larger than 20 μm. However, as illustrated in Figure 14b, the addition of a vertical dam has an overall negative impact on the removal rate. Specifically, in Case C1, where the dam is positioned immediately after the weir, the removal rate decreases for all particles smaller than approximately 30 μm, although a slight improvement is observed for larger particles. In Case C2, where the dam is located farther downstream from the weir, the removal rate declines across nearly all inclusion sizes. On average, assuming equal weighting across all inclusion sizes, Cases C1 and C2 reduce the IRR by approximately 1% and 8%, respectively.

These findings contrast with the RTD analysis results presented in the previous section, where Case C2 appeared to perform best in terms of reducing the dead zone and increasing the plug flow volume, as well as the plug-to-dead and plug-to-mixed volume ratios.

**Figure 15:**
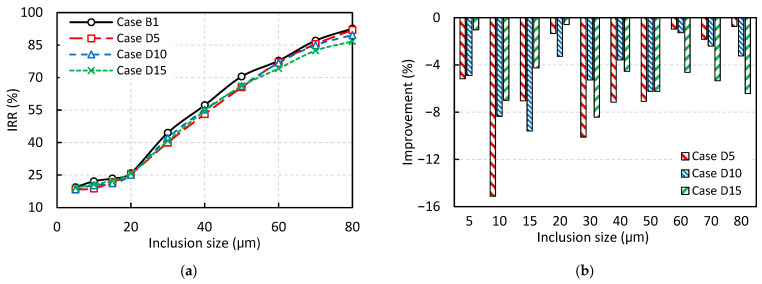
The effect of the horizontal baffle on inclusion removal rate (IRR) based on DPM analysis: (**a**) IRR, and (**b**) Percentage improvement in IRR compared to the base case without baffle (Case B1).

Figure 15 illustrates the effect of adding a horizontal baffle at the end wall of the tundish on the IRR, based on the DPM analysis. The overall trend in removal rate, shown in Figure 15a as a function of inclusion size, is consistent with that observed in Figure 13 and Figure 14. That is, the inclusion removal rate increases with inclusion size, with a more pronounced removal rate for particles larger than 20 μm. However, as shown in Figure 15b, a key observation is that the inclusion removal rate decreases in all cases with a horizontal baffle, regardless of particle size, indicating a negative impact of the horizontal baffle on tundish performance.

These findings again contrast with the RTD analysis results presented earlier in Figure 11, where Cases D5 and D10 were shown to have reduced dead zone volume and increased plug flow volume and plug-to-dead volume ratio, features typically known to be associated with improved tundish performance in terms of inclusion removal rate. Nonetheless, the DPM analysis demonstrates that all configurations involving a horizontal baffle lead to reduced IRR, thereby questioning the reliability of RTD-based metrics in fully assessing tundish performance in terms of inclusion removal efficiency.

**Figure 16:**
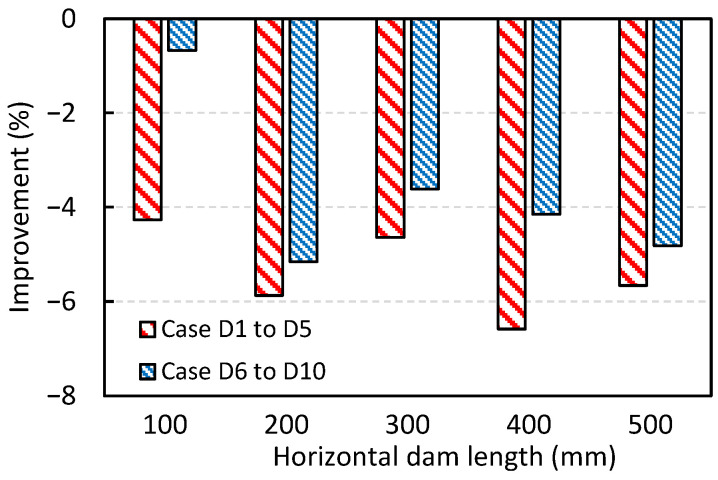
The effect of the horizontal baffle length on improvement of average IRR based on DPM analysis compared to the base case without baffle (Case B1).

Figure 16 shows the effect of horizontal baffle length on the average IRR across all inclusion sizes, compared to the base case without a baffle (i.e., Case B1). The results indicate that the presence of a horizontal baffle reduces the IRR, regardless of its length. Another important observation is that, when comparing the DPM results with the RTD analysis results presented in Figure 11, no clear correlation is found between the IRR and the reduction in dead zone volume, or the increases in plug flow volume, plug-to-dead ratio, and plug-to-mixed ratio. In other words, although the RTD metrics in Figure 11 suggest that a longer baffle (as in Cases D1 to D10) might enhance performance, the DPM results indicate that a longer baffle may actually have a detrimental effect.

### 4.4. Heat Transfer Analysis

The DPM analysis results indicated that inclining the side walls is a promising method for enhancing the inclusion removal rate, while maintaining constant tundish volume and theoretical residence time. However, this modification increases the top surface area, which may lead to greater heat loss from the melt and affect the temperature distribution within the tundish.

**Figure 17:**
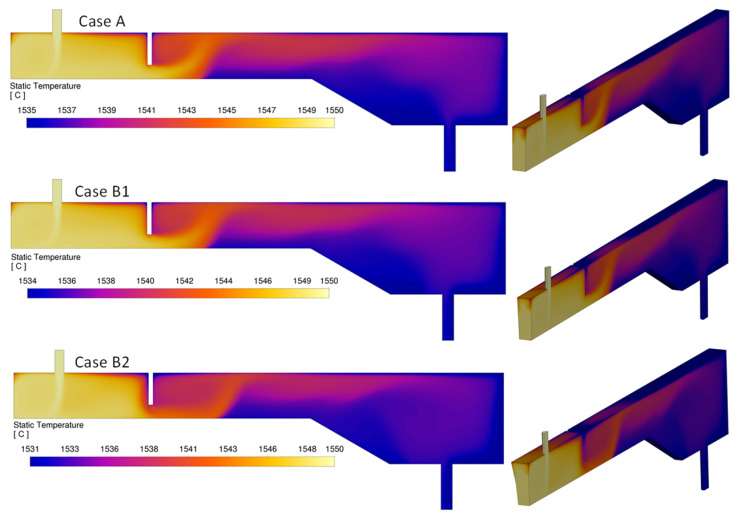
The effect of side-wall inclination on temperature distribution.

Figure 17 shows the temperature distribution for three different cases: Case A with vertical side walls, Case B1 with constant-angle inclined side walls, and Case B2 with variable-angle inclined side walls. The results suggest that side wall inclination has no significant effect on the overall temperature distribution. A well-mixed region is observed in the inlet region before the weir, followed by an upward flow immediately downstream, driven by buoyancy. A low-temperature region also appears between the point where the melt rises downstream of the weir and the outlet nozzle.

The main difference among the cases lies in the average outlet temperature: approximately 1535.6 °C for Case A, 1534.3 °C for Case B1, and 1531.0 °C for Case B2. This decrease is primarily attributed to increased heat loss from the top surface, as detailed in Table 3. Care should be taken when analyzing the outlet temperatures. Although the differences between outlet temperatures in different cases are not substantial, their true significance becomes apparent when comparing the rate of heat loss. Total heat loss increases by 8.7% in Case B1 compared to Case A, accompanied by an approximately 8% improvement in IRR, based on the DPM analysis. For Case B2, the heat loss increases by 32.0% relative to Case A, while the inclusion removal rate improves by about 19% across all inclusion sizes (5 μm to 80 μm).

### 4.5. Economic Assessment

A rough estimation of energy consumption was conducted to assess whether the improvement in IRR achieved using the new tundish design with variable-angle side walls (Case B2) is financially justifiable compared to the original design with vertical walls (Case A). As shown in Table 3, the 19% improvement in IRR comes at the cost of 32% higher heat loss from the tundish.

Considering that each production run lasts approximately 45 min, the additional energy consumed using Case B2 compared to Case A is calculated as follows:

$$Q_{extra}=\left(11894-9013\right)\times \frac{45}{60}\times 0.001=2.16\;kWh$$

Assuming a mass flow rate of 50 kg.min^−1^ (inlet boundary condition) over 45 min, the total processed melt is 2250 kg. The energy required to raise the melt from ambient temperature (25 °C) to its liquidus temperature (1400 °C), considering a specific heat (*c_p_*) of 0.75 kJ.kg^−1^.K^−1^, is:

$$Q_{sensible}=mc_{p}\Delta T=2250\times 0.75\times \left(1400-25\right)/3600=644\;kWh$$

The latent heat required for melting, considering a latent heat of fusion (Δ*H*) of 270 kJ.kg^−1^, is:

$$Q_{latent}=m\Delta H=2250\times 270/3600=169\;kWh$$

For metallurgical considerations, the melt must be superheated to approximately 150 °C above the liquidus temperature. The energy required to provide the superheat (*Q_sh_*) is calculated as follows:

$$Q_{sh}=mc_{p}(T_{sh}-T_{liquidus})=2250\times 0.75(1550-1400)/3600=70\;kWh$$

Neglecting the energy required to maintain the melt temperature in the furnace during the process run, the approximate total energy for one production run is:

$$Q_{tot}=Q_{sensible}+Q_{latent}+Q_{sh}=883\;kWh$$

Thus, the additional energy required due to the new design (*Q_extra_*) is less than 0.3% of the total energy consumed per run (*Q_tot_*), making its economic impact negligible. It should also be emphasized that inclusions are a major source of defects in the final deposit; thus, achieving a 19% improvement in inclusion removal through a passive, economically feasible technique such as variable-angle inclined side walls represents a substantial enhancement to the process design.

### 4.6. Relation Between RTD-Based Metrics and Inclusion Particles Behavior

In this section, Pearson correlation is used as a linear statistical method to evaluate the relationship between RTD-based metrics and inclusion removal rates obtained from DPM analysis. The correlation coefficient, *r*, ranges from −1 to 1 and is calculated using the following equation:

(19) $$r=\frac{\sum_{i=1}^{n}\left(x_{i}-\bar{x})(y_{i}-\bar{y}\right)}{\sqrt{\sum_{i=1}^{n}{\left(x_{i}-\bar{x}\right)}^{2}}\sqrt{\sum_{i=1}^{n}{\left(y_{i}-\bar{y}\right)}^{2}}},$$

where *x* and *y* represent RTD-based metric (e.g., plug-to-dead volume ratio) and the IRR, respectively. The sign of *r* indicates the direction of the relationship, while its magnitude reflects the strength, with absolute values greater than 0.6 considered strong [55]. The following RTD-based metrics are examined:

- Dead volume fraction (*V_d_*)
- Plug flow volume fraction (*V_p_*)
- Mixed flow volume fraction (*V_m_*)
- Plug-to-dead volume ratio
- Plug-to-mixed volume ratio.

To provide a clearer analysis, simulation results are categorized into three groups based on the type of FCDs and geometric modifications:

1. Group I: Cases A, B1, and B2 (effect of side-wall inclination)
2. Group II: Cases B1, C1, C2 (effect of vertical dam insertion)
3. Group III: Cases B1, D5, D10, D15 (effect of horizontal baffle installation)

**Figure 18:**
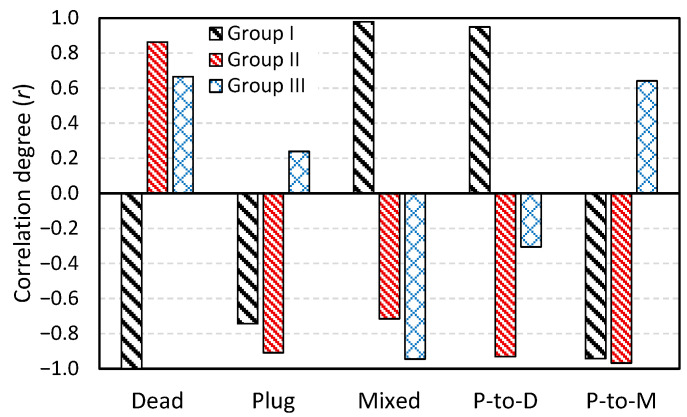
The degree of correlation between RTD-based metrics and inclusion removal rate (IRR) for different groups: Group I—Cases A, B1, and B2; Group II—Cases B1, C1, and C2; and Group III—Cases B1, D5, D10, and D15. P-to-D and P-to-M refer to plug-to-dead and plug-to-mixed volume ratios, respectively.

The computed *r* values for each group are summarized in Table 4 and visualized in Figure 18. A key finding is that none of the RTD-based metrics show a consistent correlation sign across all three groups. In other words, for any given RTD metric, the correlation with IRR can be either positive or negative, depending on the FCD type and geometric configuration. For instance, the dead volume fraction exhibits a strong inverse correlation (*r* ≈ −1.0) with IRR in Group I, indicating that reducing dead volume significantly improves inclusion removal. However, in Groups II and III, the dead volume fraction shows a strong direct correlation (*r* > 0.6) with IRR. The opposite trend is observed for the plug-to-dead ratio, which is positively correlated with IRR in Group I, but negatively correlated in Groups II and III.

These findings indicate that it is not possible to define a single, consistent correlation between RTD-based metrics and IRR across all geometric configurations. The direction and strength of the correlation vary depending on the type of FCD and the type of geometric modification employed. In other words, the RTD behavior and its influence on inclusion removal change with the specific design features implemented in the tundish. A similar conclusion was reached by another research group [20] in a recent isothermal study that aimed to correlate RTD characteristics with inclusion behavior in tundishes; however, using a different approach based on solving transport equations in a Eulerian framework, rather than tracking individual particles as conducted in the present study. This reinforces the importance of conducting combined fluid flow and particle transport analyses tailored to each tundish design, rather than relying on general RTD-based trends.

## 5. Conclusions

Optimizing tundish flow behavior is essential for minimizing non-metallic inclusions and advancing cleaner steel production. This approach plays a key role in achieving more sustainable and resource-efficient steelmaking, especially as the industry increasingly relies on recycled materials. A series of CFD simulations were performed to re-examine the flow characteristics of tundishes used in different steelmaking processes. The primary objective was to redesign an existing tundish and identify a passive, simple, and practical method to improve inclusion removal rate (IRR), using residence time distribution (RTD) and discrete phase model (DPM) analyses. RTD analysis was employed to derive key metrics such as dead zone and plug flow volume fractions, as well as plug-to-dead and plug-to-mixed volume ratios to evaluate tundish performance. DPM analysis was used to track the trajectories of inclusions of various sizes as they moved through the tundish. Three different modification techniques were investigated: adding a vertical dam, adding a horizontal baffle, and altering the tundish geometry by inclining the side walls, in order to assess their effects on inclusion removal efficiency. The main findings of this study are summarized as follows:

- Temperature variations inside a tundish due to heat losses induce buoyancy-driven flows, which significantly affect the fluid flow and RTD behavior. These temperature-induced effects can potentially enhance RTD characteristics by reducing the likelihood of short-circuiting, minimizing dead zone volume, and increasing plug flow volume.
- The DPM analysis results showed that inclining the side walls, using a constant angle in Case B1 and a variable angle in Case B2, was the most effective approach for increasing the IRR. Average improvements of 8% and 19% were observed, respectively, across all inclusion sizes from 5 to 80 μm, compared to the base case with vertical side walls (Case A). However, this improvement in removal rate comes at the cost of increased heat loss to the surroundings, with total heat loss rising by 8.7% in Case B1 and 32.0% in Case B2 relative to Case A. This increase in heat loss is attributed to the larger exposed top surface area in the modified cases.
- Adding a vertical dam (Cases C1 and C2), regardless of its position, reduced the average IRR for nearly all inclusion sizes studied (5 to 80 μm), according to the DPM analysis. However, in Case C1, where the dam was located immediately after the weir, an improvement in IRR was observed for inclusions larger than 30 μm. Meanwhile, the RTD analysis indicated that the addition of a vertical dam reduced the dead zone volume, while increasing the plug flow volume, the plug-to-dead ratio, and the plug-to-mixed ratio, factors that would typically be expected to enhance IRR.
- Adding a horizontal baffle (Cases D1 to D15), regardless of its position or length, reduced the IRR across all inclusion sizes, according to the DPM analysis. For instance, in Case D5, the maximum reduction in removal rate was 15% for inclusions of 10 μm, while the average reduction across all inclusion sizes was 5.7%. Once again, the RTD analysis results contrasted with the DPM findings, showing that adding a horizontal baffle decreased the dead zone volume while increasing the plug flow volume and plug-to-dead ratio.
- Pearson correlation analysis revealed that RTD-based metrics do not exhibit a consistent relationship with IRR across different tundish configurations. The direction and strength of correlation varied significantly depending on the type of FCD and geometric modification. For example, dead volume fraction showed a strong negative correlation with IRR in tundishes with inclined side walls but a strong positive correlation in those with vertical dams or horizontal baffles.

Overall, the findings of the present study underscore the need for case-specific analyses when evaluating tundish performance in inclusion removal. Generalized RTD-IRR correlations and RTD-based metrics (such as plug flow, mixed flow, dead zone, and their ratios) may lead to misleading interpretations, highlighting the importance of detailed DPM analysis for a better understanding of inclusion behavior within tundishes.

## Figures and Tables

**Table 1:** Detailed geometric characteristics of all simulated cases.

Case ID	L1	L2	L3	Side Wall
Case A	×	×	×	Vertical
Case B1	×	×	×	Constant inclination
Case B2	×	×	×	Variable inclination
Case C1	×	×	500	Constant inclination
Case C2	×	×	100	Constant inclination
Cases D1 to D5	100 (to 500)	100	×	Constant inclination
Cases D6 to D10	100 (to 500)	200	×	Constant inclination
Cases D11 to D15	100 (to 500)	300	×	Constant inclination

**Table 2:** RTD parameters and calculated volume fractions for Case A and Case C2.

Case	*t_r,th_* (s)	*θ_avg_*	*t_min_* (s)	*t_peak_* (s)	*V_d_* (%)	*V_p_* (%)	*V_m_* (%)
A—Isothermal	302.1	0.605	50.5	113.0	47	27	26
A—Non-isothermal	302.1	0.782	70.0	134.5	30	33	37
C2—Isothermal	301.5	0.804	71.5	146	27	36	37
C2—Non-isothermal	301.5	0.837	81.0	151.5	23	39	38

**Table 3:** Summary of heat transfer characteristics and inclusion removal enhancement for Cases A, B1, and B2, highlighting the effect of side-wall inclination on tundish performance.

Case	Outlet Temp. (°C)	Heat Loss (W)			Change in Heat Loss	Inclusion Removal Enhancement
		Top	Side	Total		
A	1535.6	8123	890	9013	-	-
B1	1534.3	8923	876	9799	8.7%	8%
B2	1531.0	11,034	860	11,894	32.0%	19%

**Table 4:** Summary of Pearson correlation results.

**Group I**	***V_d_*** **(%)**	***V_p_* (%)**	***V_m_* (%)**	**Plug-to-Dead**	**Plug-to-Mixed**	**Average IRR**
Case A	30	34	37	1.15	0.92	49.1
Case B1	27	35	38	1.30	0.91	52.1
Case B2	23	32	45	1.37	0.71	56.7
*r*	−1.0	−0.74	0.98	0.95	−0.94	NA
**Group II**	***V_d_* (%)**	***V_p_* (%)**	***V_m_* (%)**	**Plug-to-Dead**	**Plug-to-Mixed**	**Average IRR**
Case B1	27	35	38	1.30	0.91	52.1
Case C1	37	28	34	76	0.82	52.8
Case C2	23	39	38	1.67	1.00	49.9
*r*	0.86	−0.91	−0.72	−0.93	−0.97	NA
**Group III**	***V_d_* (%)**	***V_p_* (%)**	***V_m_* (%)**	**Plug-to-Dead**	**Plug-to-Mixed**	**Average IRR**
Case B1	27	35	38	1.30	0.91	52.06
Case D5	16	38	46	2.39	0.82	49.76
Case D10	16	40	44	2.55	0.90	49.98
Case D15	22	26	51	1.18	0.52	49.28
*r*	0.66	0.24	−0.95	−0.31	0.64	NA

## Data Availability

The original contributions presented in this study are included in the article. Further inquiries can be directed to the corresponding author.
